# Supplementary figures and images for: Mutual influence between language and perception in multi-agent communication games
Source: PLoS Comput Biol. 2022 Oct 31;18(10):e1010658. doi: 10.1371/journal.pcbi.1010658 (PMC9648844; doi:10.1371/journal.pcbi.1010658)

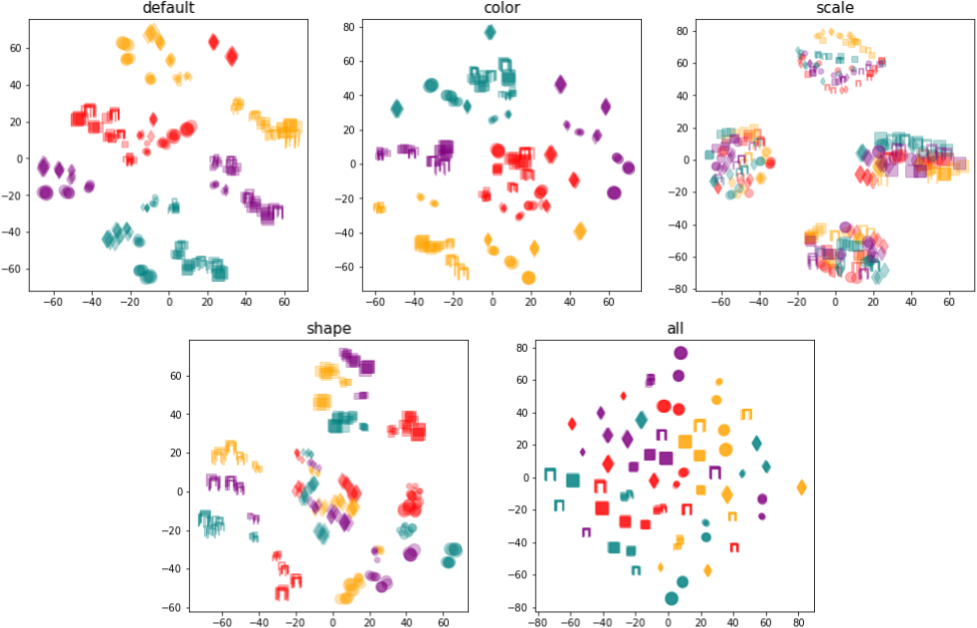

Supplement: S1 Fig — The four color and scale values are given by the four marker colors and marker sizes, while the following mapping from object shape to marker shape is used: (cube, sphere, cylinder, ellipsoid) → (square, circle, square cap (⊓), rhombus (⋄)). t-SNE embeddings were calculated on a data subset of 100 random examples per class (6400 data points) using a perplexity of 100, and 2000 iterations. Plotted are the embeddings for 5 random examples per class. In the default and color conditions, clusters form around color values, in the shape condition around shape values, and in the scale condition around scale values. The complex similarity relationships in the all condition do not fall into clear clusters in two dimensions. (TIF) [file pcbi.1010658.s001.tif]

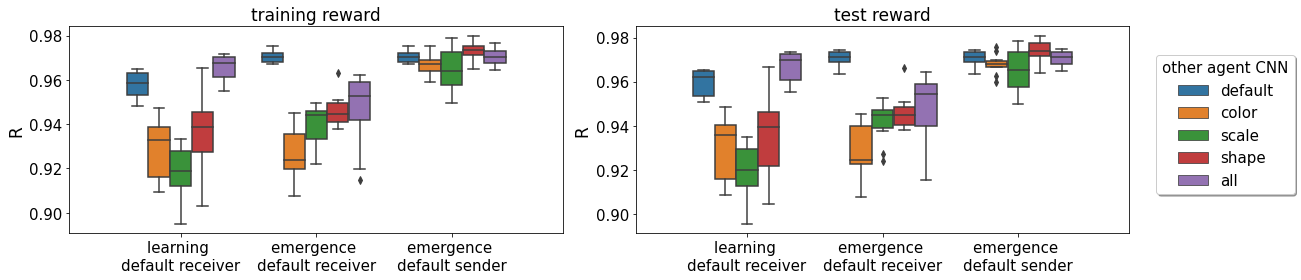

Supplement: S3 Fig — Shown are boxplots of training and test rewards in the language learning and language emergence scenarios, when studying the influence of differences in language on perception. The plots are generated from the results across ten runs each for communication partners with different perceptual biases (color-coded), always in combination with a default agent. In the language learning scenario, the sender (vision and language module) is fixed and we study the effects on the default receiver, that is learning the language. In the language emergence scenario, we consider the two cases that a default receiver is paired with different senders, and that a default sender is paired with different receivers. (TIF) [file pcbi.1010658.s003.tif]

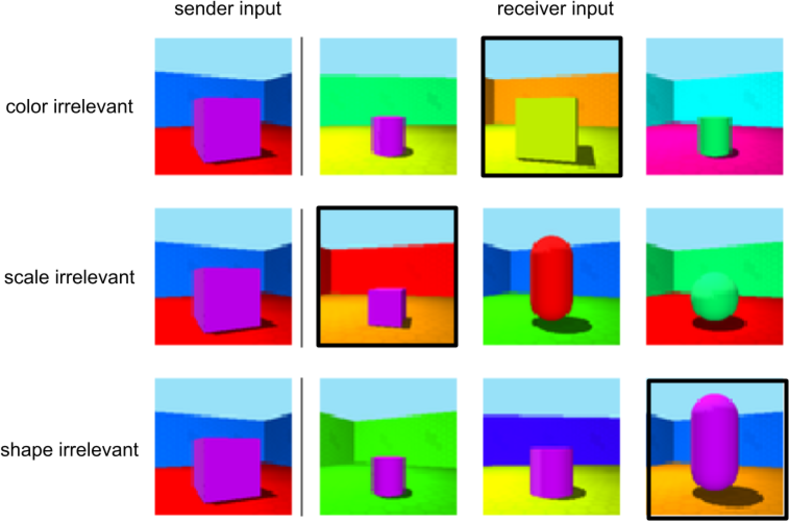

Supplement: S4 Fig — In our control experiments for the evolutionary analysis, we vary which attributes are relevant to the communication game. Always two of the attributes color, scale, and shape are relevant, i.e. one attribute is not relevant. For the irrelevant attribute, sender and receiver target may have different values. Shown are example inputs for different relevance conditions: color irrelevant (top row), scale irrelevant (middle row), and shape irrelevant (bottom row). The receiver target for each condition is marked by a black box. (TIF) [file pcbi.1010658.s004.tif]

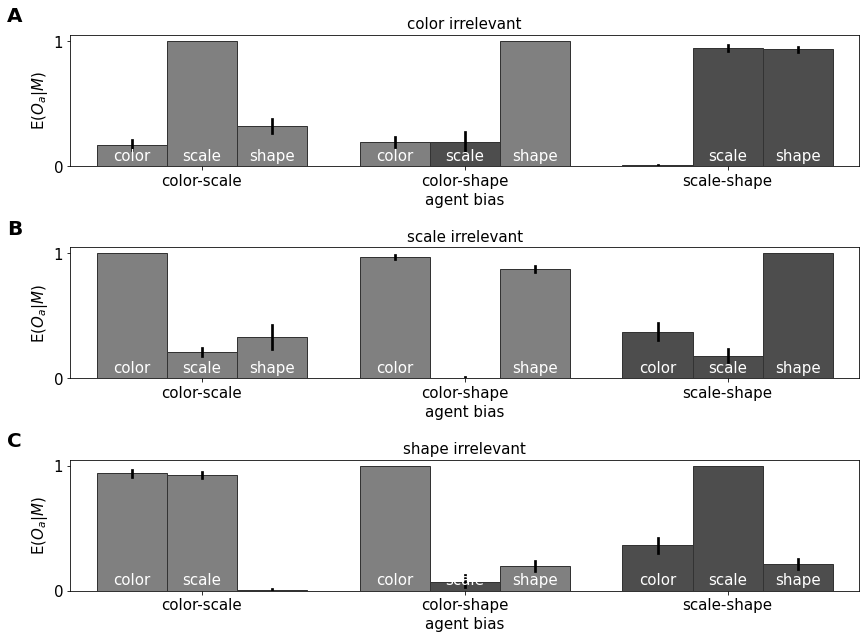

Supplement: S5 Fig — Shown are the effectiveness scores per attribute when combining a sender and a receiver with the same mixed bias. The agents’ bias is given on the x-axis, the score on the y-axis, and the attribute for which the score is calculated is indicated by the bar labels. Bars of enforced attributes are dark gray. Results are shown for the three different relevance conditions: (A) color irrelevant, (B) scale irrelevant, (C) shape irrelevant. We report means and bootstrapped 95% CIs of twenty runs each. Again, the differences in visual perception systematically influence the emerging language. The scores further show that only visual biases for task-relevant attributes are reflected in the language. (TIF) [file pcbi.1010658.s005.tif]
