# Supplementary material for: Mutual influence between language and perception in multi-agent communication games
Source: PLoS Comput Biol. 2022 Oct 31;18(10):e1010658. doi: 10.1371/journal.pcbi.1010658 (PMC9648844; doi:10.1371/journal.pcbi.1010658)
Supplement: S1 Table — (PDF) [file pcbi.1010658.s006.pdf]

S1 Table. Performance of biased-default agent combinations when only the language modules are trained.

		color	scale	shape	all
S biased, R default	train reward	0.919 ± 0.008	0.914 ± 0.009	0.944 ± 0.006	**0.951** ± 0.005
	test reward	0.922 ± 0.008	0.917 ± 0.009	0.947 ± 0.006	**0.954** ± 0.005
	$\overline{E(O_a, M)}$	0.594 ± 0.015	0.584 ± 0.017	0.656 ± 0.019	**0.688** ± 0.015
R biased, S default	train reward	0.945 ± 0.013	0.959 ± 0.003	**0.965** ± 0.005	0.960 ± 0.003
	test reward	0.947 ± 0.014	0.962 ± 0.003	**0.966** ± 0.005	0.961 ± 0.004
	$\overline{E(O_a, M)}$	0.666 ± 0.020	0.706 ± 0.015	**0.742** ± 0.015	0.689 ± 0.014

Shown are training rewards, test rewards, and average effectiveness across attributes for sender-receiver (S-R) pairs consisting of one biased and one DEFAULT agent. Reported are means and bootstrapped 95% CIs of twenty runs per condition. The best values across conditions are highlighted.
