# Supplementary material for: Mutual influence between language and perception in multi-agent communication games
Source: PLoS Comput Biol. 2022 Oct 31;18(10):e1010658. doi: 10.1371/journal.pcbi.1010658 (PMC9648844; doi:10.1371/journal.pcbi.1010658)
Supplement: S1 Appendix — (PDF) [file pcbi.1010658.s007.pdf]

Entropy analysis between target objects, messages, and selections

The schema for a three-way information-theoretic analysis of the relation between target objects O, messages M, and selected objects S, is depicted in Fig 1. Quantifying all terms requires generalized definitions of (conditional) mutual information and conditional entropy for three random variables. The mutual information between three variables, also known as interaction information, is defined as

$$I(X, Y, Z) = I(X, Y) - I(X, Y \mid Z)\,,$$

where the conditional mutual information is the expected mutual information between X and Y given Z:

$$I(X, Y \mid Z) = \sum_{z \in Z} \sum_{y \in Y} \sum_{x \in X} p(x, y, z) \log \frac{p(z)p(x, y, z)}{p(x, z)p(y, z)}\,.$$

The conditional entropy of X given Y and Z quantifies the amount of uncertainty that remains about X when knowing Y and Z

$$H(X \mid Y, Z) = - \sum_{z \in Z,\ y \in Y,\ x \in X} p(x, y, z) \log \frac{p(x, y, z)}{p(y, z)}\,.$$

Our analyses show that the mutual information between objects and selections given messages is approximately zero in all experiments, $I(O, S \mid M) \approx 0$. In other words, the shared information between target and selection is fully predicted by the messages. The symmetry between sender (objects-messages) and receiver (messages-selections) analysis can also be identified in this more general framework in terms of the following relationships: $H(O \mid M, S) \approx H(S \mid O, M)$ and $I(O, M \mid S) \approx I(M, S \mid O)$.

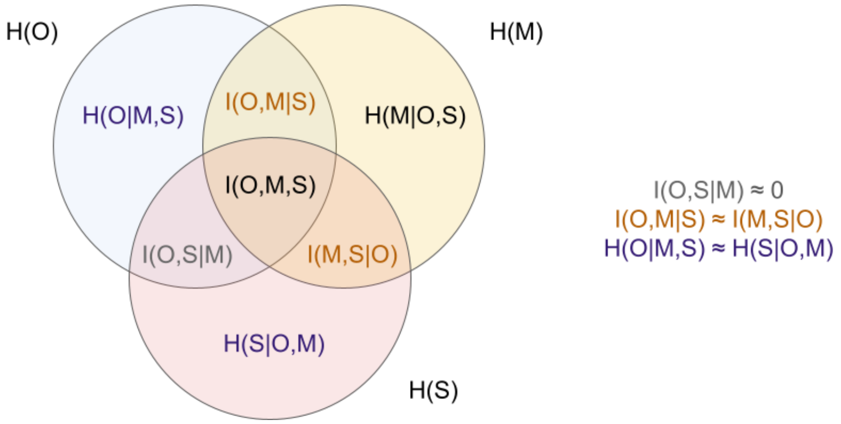

Fig 1. Schema of the information in the target objects, O, the corresponding messages, M, and objects selected by the receiver, S. H denotes entropy and I mutual information. Note, the schema is not an actual set-theoretic representation and serves illustrative purposes only.
