# Supplementary material for: Mutual influence between language and perception in multi-agent communication games
Source: PLoS Comput Biol. 2022 Oct 31;18(10):e1010658. doi: 10.1371/journal.pcbi.1010658 (PMC9648844; doi:10.1371/journal.pcbi.1010658)
Supplement: S2 Appendix — (PDF) [file pcbi.1010658.s008.pdf]

Increasing vocabulary size and number of distractors

Fig 1 shows the effectiveness scores for different vocabulary sizes and numbers of distractors across ten runs per condition. For $|V| = 4$ (top row) increasing the number of distractors does not increase effectiveness. Given this limited vocabulary size, the communicative content does not improve when more distractors are used. Increasing the vocabulary size to $|V| = 8$ or $|V| = 12$ (center and bottom rows) makes the task easier and allows the agents to find better protocols, which is reflected in higher effectiveness scores (and test rewards, not shown here). Increasing the number of distractors in addition to the vocabulary size (right column) can further increase the average effectiveness for some conditions. Although average effectiveness increases with a larger vocabulary size in the DEFAULT condition, average effectiveness in the ALL condition is still significantly higher for vocab size $|V| = 8$ and $|V| = 12$ and either number of distractors (lower bounds of bootstrapped 95% CIs for differences in means > 0.020); and so are the test rewards (not shown here). So, also when nudged to communicate more information about each attribute, ALL agents develop better protocols than DEFAULT agents.

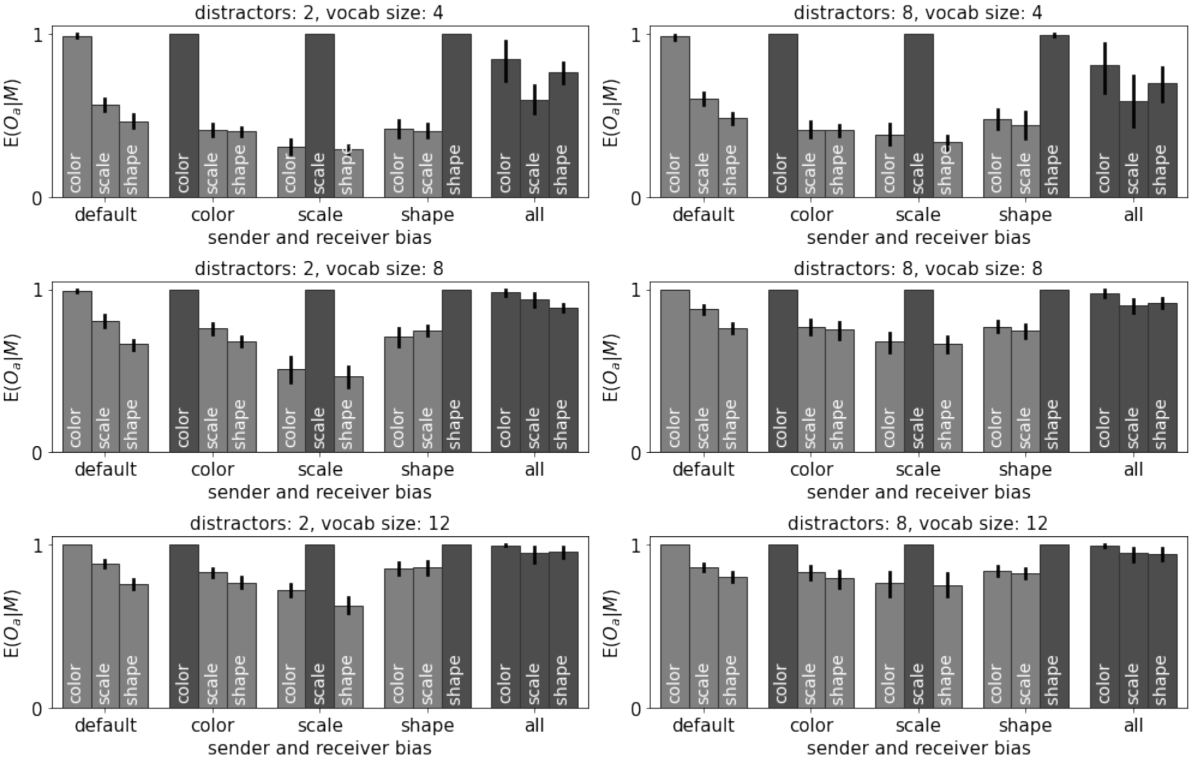

Fig 1. Effectiveness per attribute for different vocabulary sizes ($|V| \in \{4, 8, 12\}$), and different numbers of distractors ($k \in \{2, 8\}$). Sender-receiver pairs with the same bias play the reference game, and only the language module weights are trained. The bars are labeled with the attribute a used for calculating $E(O_a|M)$, with attributes enforced via label smoothing in dark gray. We report means and bootstrapped 95% CIs calculated from ten runs each.
