# Supplementary material for: Mutual influence between language and perception in multi-agent communication games
Source: PLoS Comput Biol. 2022 Oct 31;18(10):e1010658. doi: 10.1371/journal.pcbi.1010658 (PMC9648844; doi:10.1371/journal.pcbi.1010658)
Supplement: S3 Appendix — (PDF) [file pcbi.1010658.s009.pdf]

Control simulations without classification loss

In these control simulations, we study the influence of language on perception when the agents are trained on the reference game but not the classification task. We rerun the original simulations without classification loss for the DEFAULT, ALL, and SCALE condition. The latter serves as a representative of the single-attribute bias conditions. The classification loss stabilizes training and allows for a higher learning rate. Without the classification loss, we reduce the learning rate to 0.0001 and increase the number of epochs to 50 in the language learning scenario and 250 in the language emergence scenario. Apart from that, we use the original hyperparameters and training procedure. The average test rewards for the language learning scenario lie between 0.921–0.967 and for the language emergence scenarios between 0.905–0.937.

Fig. 1 (top row) shows the visual biases of the DEFAULT agent after training, for communication partners with different visual biases (color-coded). Overall the resulting biases show the same patterns as in the original simulations (see Fig. 7 in the main text). We can confirm the main finding that language influences perception. If the DEFAULT agent communicates with a biased agent (e.g. SCALE), the bias of the communication partner leads to an increase in the RSA score of the corresponding attribute (scale).

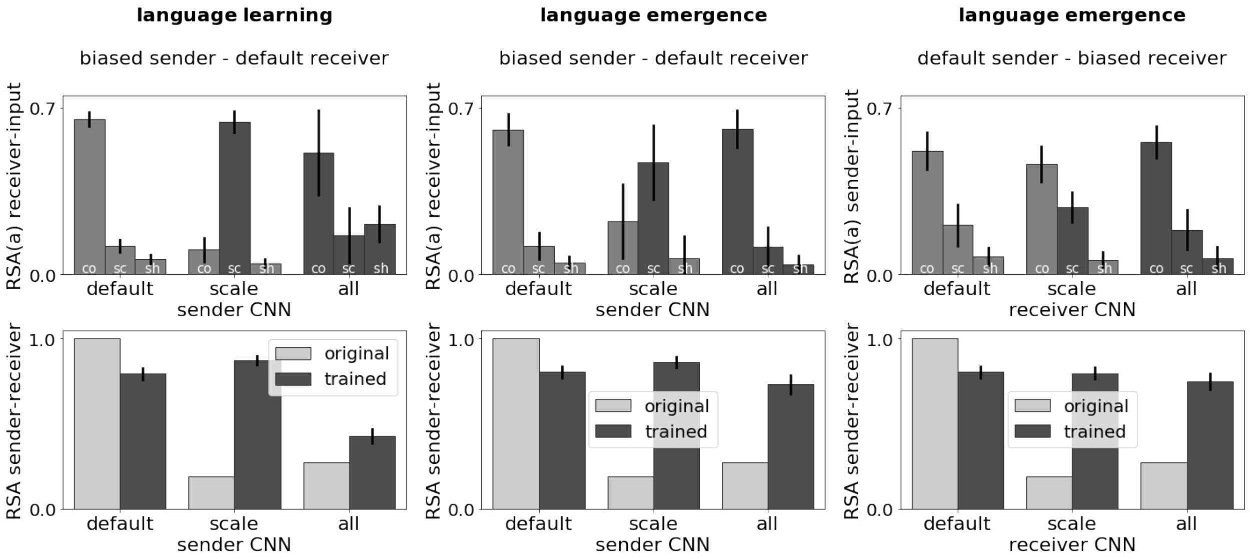

Fig 1. Influence of linguistic biases on perception. Shown are the effects of language learning and language emergence on a DEFAULT agent, when paired with agents of different visual bias conditions. The left column covers the language learning scenario with a DEFAULT receiver, the central column the language emergence scenario with a DEFAULT receiver, and the right column the language emergence scenario with a DEFAULT sender. In the language learning scenario, the sender's weights (and therefore also the language) are entirely fixed. In the language emergence scenario, both agents are trained and the language emerges. The visual bias of the communication partner is shown on the x-axis. The top row shows the RSA scores between the DEFAULT agent's visual representations and each object attribute—indicated by the bar label (*co*: color, *sc*: scale, *sh*: shape)—after training. The bottom row shows the RSA scores between the visual representations of the DEFAULT agent and those of its communication partner before (light gray) and after (dark gray) training. Reported are means and bootstrapped 95% CIs of ten runs each.

Comparing the two figures, the classification loss seems to have a moderating effect on the visual representations. Without classification, the linguistic biases are more strongly reflected in the visual biases. In the language learning scenario, this effect can be observed for the DEFAULT sender (color bias) and the SCALE sender. The RSA scores for the biased attribute increase while the other RSA scores decrease. In the language emergence scenario, this effect can be observed from an increase in RSA_{color} in most conditions. One could assume that the agents start out with a strong color bias (DEFAULT agents) and keep that bias because the effect of communication is weaker without classification. However, the language learning scenario shows that this is not the case. Rather, it seems that all agents increasingly focus on color information. The color bias must stem from the input representation or the CNN architecture and not the classification objective. Without classification, the induced biases can revert to a color bias, which then dominates the conversation and as a result also the changes in visual representations. For example, the color bias becomes more prominent in interactions between DEFAULT and ALL agents. At the same time, RSA_{shape} decreases across simulations, as shape information is no longer enforced by the classification task and is not the focus of the DEFAULT or SCALE agent. While shape information does originally play a role for the ALL agent, it is mostly overwritten by color information in the language emergence process. In conclusion, the classification loss constrains the visual representations to also capture differences between the values of attributes that do not conform to the linguistic bias.

As the agents discriminate between fewer objects in communication than in classification (communication is less optimal than classification), the visual representations contain less information if they only serve communication. Therefore, an increase in the overall RSA scores after training can only be observed if training on the classification task continues.
