# Supplementary material for: Mutual influence between language and perception in multi-agent communication games
Source: PLoS Comput Biol. 2022 Oct 31;18(10):e1010658. doi: 10.1371/journal.pcbi.1010658 (PMC9648844; doi:10.1371/journal.pcbi.1010658)
Supplement: S4 Appendix — (PDF) [file pcbi.1010658.s010.pdf]

Grid search for mixed-bias agents

We conducted a grid search to generate comparable mixed-bias agents. We pretrained CNNs enforcing always two attributes: color and scale, color and shape, or scale and shape. The goal of our search was to identify a network for each condition, such that 1) biases for enforced attributes are strong, 2) biases for enforced attributes are approximately equally strong within and across networks, 3) biases for not enforced attributes are approximately zero, and 4) achieved training accuracies are reasonably high. For the grid search we varied the smoothing factor $\sigma \in \{0.6, 0.7, 0.8\}$, and used different weightings between the two enforced biases $w \in \{[0.05, 0.95], [0.10, 0.90], [0.15, 0.85], \ldots, [0.85, 0.15], [0.90, 0.10], [0.95, 0.05]\}$. We selected a network for each condition (see Table 1), by optimizing the first three criteria under the constraint of a minimum training accuracy of 0.97. For each condition, the smoothing factor 0.8 yielded the best network. The weighting parameters show that to obtain these results one must counterbalance the networks' inherent color bias, by using weaker enforcement for color than the other attribute. Biases for enforced attributes lie around 0.45, and biases for other attributes around 0.00.

Table 1. Results of the grid search across mixed-bias networks.

condition	σ	w	test r	RSA_{color}	RSA_{scale}	RSA_{shape}
color-scale	0.8	$[0.30, 0.70]$	0.996	0.444	0.483	0.000
color-shape	0.8	$[0.35, 0.75]$	1.000	0.458	-0.002	0.435
scale-shape	0.8	$[0.75, 0.25]$	0.974	-0.001	0.464	0.430

Each row shows parameters (smoothing factor σ and weighting w), test rewards (test r), and visual biases measured as RSA scores between network representations and attribute templates ($RSA_{attribute}$).

Simply using a fixed smoothing factor (e.g. $\sigma = 0.6$) and enforcing both relevant traits with equal weight yields the same qualitative but weaker quantitative results in the evolutionary analysis, compared to using the networks obtained from the grid search. Quantitative differences arise due to systematic (inherent color preference) and unsystematic (random seed) imbalances between network biases. For example, in a game where color and shape are relevant, COLOR-SHAPE agents should achieve particularly high rewards. But if a COLOR-SHAPE agent has a very strong color but weak shape bias, and a SCALE-SHAPE agent has a comparatively stronger shape bias, combining the two agents may result in similarly high rewards. The grid search allows us to eliminate such confounding effects.
