# Supplementary material for: Mutual influence between language and perception in multi-agent communication games
Source: PLoS Comput Biol. 2022 Oct 31;18(10):e1010658. doi: 10.1371/journal.pcbi.1010658 (PMC9648844; doi:10.1371/journal.pcbi.1010658)
Supplement: S5 Appendix — (PDF) [file pcbi.1010658.s011.pdf]

Extension to two senders and two receivers

We test whether our results from the 2-agent setup generalize to a 4-agent setup with two senders and two receivers. We run simulations for the DEFAULT, ALL, and SCALE condition. The latter serves as a representative of the single-attribute bias conditions. The two senders always have the same perceptual bias, and so do the receivers. In general, we use the same architectures, hyperparameters, and training regime as in the original simulations, with the exception that for each batch a sender and a receiver are randomly selected for training. Because convergence speed decreases with the number of agents, we extend the training time to 250 epochs. We rerun each of the three analyses: (i) influence of perception on language, (ii) influence of language on perception, and (iii) evolutionary analysis. The reported values for senders/receivers are obtained by averaging across the two senders/receivers, and the reported values for sender-receiver pairs are obtained by averaging across all sender-receiver pairs. The results for four agents are qualitatively identical to the results with two agents. Hence, we refer the reader to the Results section in the main text for explanations.

(i) For the agents' performance on the test set, please refer to analysis (iii). The effectiveness scores are shown in Fig 1, which corresponds to Fig 6 of the 2-agent simulations in the main manuscript.

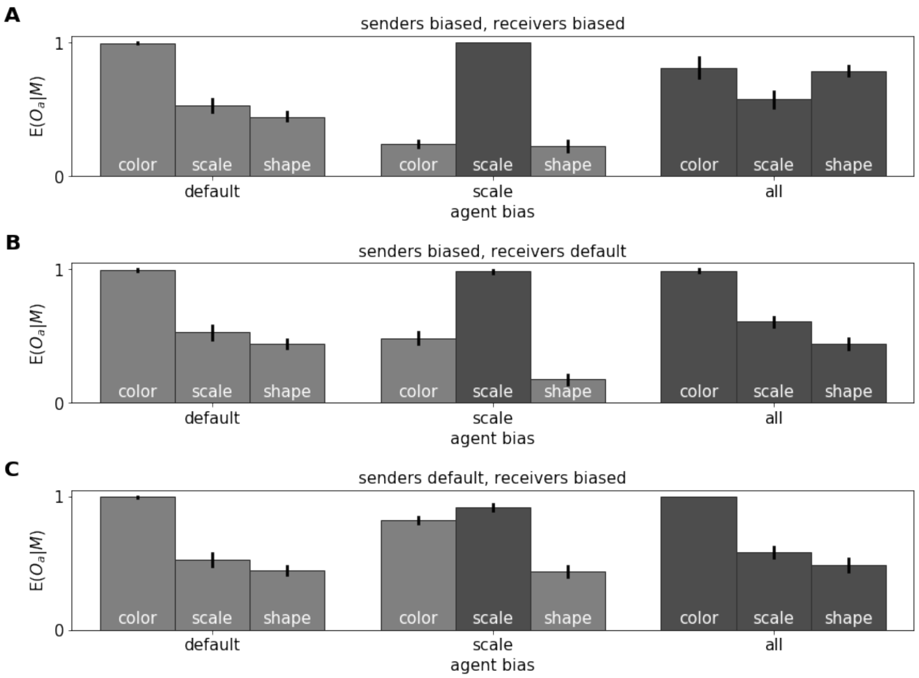

Fig 1. Effectiveness per attribute for different combinations of two senders and two receivers. Pairings are (A) senders and receivers with the same perceptual bias, (B) biased senders and DEFAULT receivers, and (C) biased receivers and DEFAULT senders. The x-axis shows the agents' perceptual biases. The bars are labeled with the attribute a used for calculating $E(O_a \mid M)$, with attributes enforced via label smoothing in dark gray. We report means and bootstrapped 95% CIs of ten runs each.

(ii) The language learning scenario does not apply to the 4-agent simulations because it tests the effects of learning a specific language on an individual. Hence, results are reduced to the language emergence scenario.

The agents achieve average rewards between 0.927 and 0.966 on the test set. The attribute-wise RSA scores are shown in Fig 2, which corresponds to Fig 7 of the 2-agent simulations in the main manuscript. In analogy to Fig 8 in the main manuscript, we calculate the difference in general RSA scores before and after training. The RSA score of the DEFAULT receiver improves from 0.439 before training to 0.553 (DEFAULT sender), 0.556 (SCALE sender), and 0.595 (ALL sender). The RSA scores of the DEFAULT sender improves from 0.439 before training to 0.574 (DEFAULT receiver), 0.600 (SCALE receiver), and 0.604 (ALL receiver).

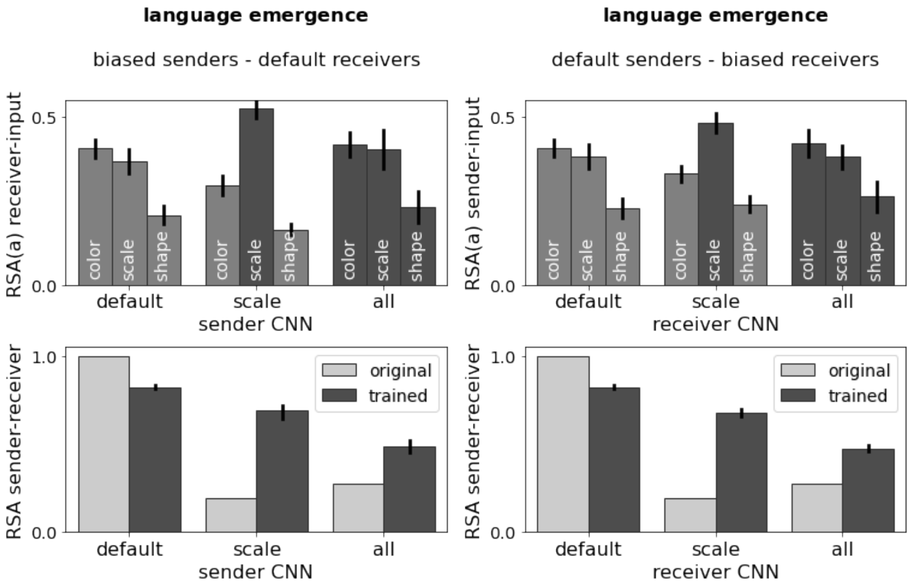

Fig 2. Influence of linguistic biases on perception. Shown are the effects of language emergence on the DEFAULT receivers when paired with biased senders (left column) and the effects on the DEFAULT senders when paired with biased receivers (right column). The visual bias of the communication partners is shown on the x-axis. The top row shows the RSA scores between the DEFAULT agents' visual representations and each object attribute—indicated by the bar label—after training. The bottom row shows the RSA scores between the visual representations of the DEFAULT agent and those of its communication partner before (light gray) and after (dark gray) training. Reported are means and bootstrapped 95% CIs of ten runs each.

(iii) The payoff matrix for different agent combinations is shown in Fig 3, which corresponds to Fig 9.A of the 2-agent simulations in the main manuscript. Again, the general patterns are comparable. Also in the 4-agent case, pairwise comparisons between the CIs in each matrix column reveal that only the evolutionary stability of the ALL bias is significant.

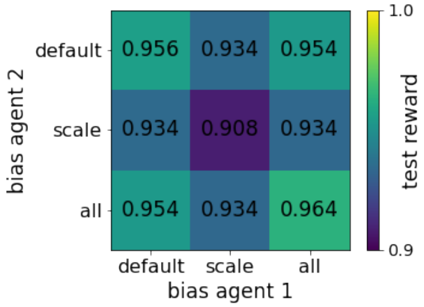

Fig 3. Mean reward on the test set for two senders and two receivers of different bias types communicating with each other. For each sender-receiver combination, we ran ten simulations. To obtain the average reward for agents of bias type t' communicating with agents of bias type t, we average the rewards of the combinations t'-senders/t-receivers and t-senders/t'-receivers, hence the matrices are symmetric. Results are shown for the basic reference game where all attributes (color, scale, shape) are relevant.

In sum, across analyses, the findings from simulations with two agents generalize to simulations with four agents.
