# Supplementary material for: Mutual influence between language and perception in multi-agent communication games
Source: PLoS Comput Biol. 2022 Oct 31;18(10):e1010658. doi: 10.1371/journal.pcbi.1010658 (PMC9648844; doi:10.1371/journal.pcbi.1010658)
Supplement: S6 Appendix — (PDF) [file pcbi.1010658.s012.pdf]

Extension to flexible-role agents

We test whether our results generalize from fixed-role agents to flexible-role agents. We run simulations for the DEFAULT, ALL, and SCALE condition. The latter serves as a representative of the single-attribute bias conditions. The sender and the receiver in the original simulations have the same architecture, apart from an additional dense layer in the sender model. Our flexible-role agent therefore uses the same model architecture as the sender. If it is used as a receiver, the additional layer remains unused, and the hidden state of the language module is initialized with a zero vector. The vision module is always used to process the input image(s) and the language module is either used to generate or to interpret a message, depending on the current task of the agent. Note, that this setup does not guarantee that both agents will converge on sending the same messages, and to the best of our knowledge there is no trivial way to enforce such behavior. We use the same hyperparameters and training regime as in the original simulations, with the exception that for each batch one of the agents is randomly assigned the role of sender and the other agent the role of receiver. We rerun each of the three analyses: (i) influence of perception on language, (ii) influence of language on perception, and (iii) evolutionary analysis. Across all analyses, simulations with flexible-role agents yield the same qualitative results as simulations with fixed-role agents. Hence, we refer the reader to the Results section in the main text for explanations.

(i) For the agents' performance on the test set, please refer to analysis (iii). The effectiveness scores are shown in Fig 1, which corresponds to Fig 6 of the fixed-role agent simulations in the main manuscript. In part A, the effectiveness scores are averaged across both (biased) agents for each run. Parts B and C show the results for the combination of one biased and one DEFAULT agent. As mentioned above, the agents do not necessarily speak the same language, hence we analyze the effectiveness scores for the biased agent (B) and the DEFAULT agent (C) separately. The effectiveness scores for the SCALE-DEFAULT combinations show that the biases of both agents are reflected in the protocol (color bias for the DEFAULT agent and scale bias for the SCALE agent).

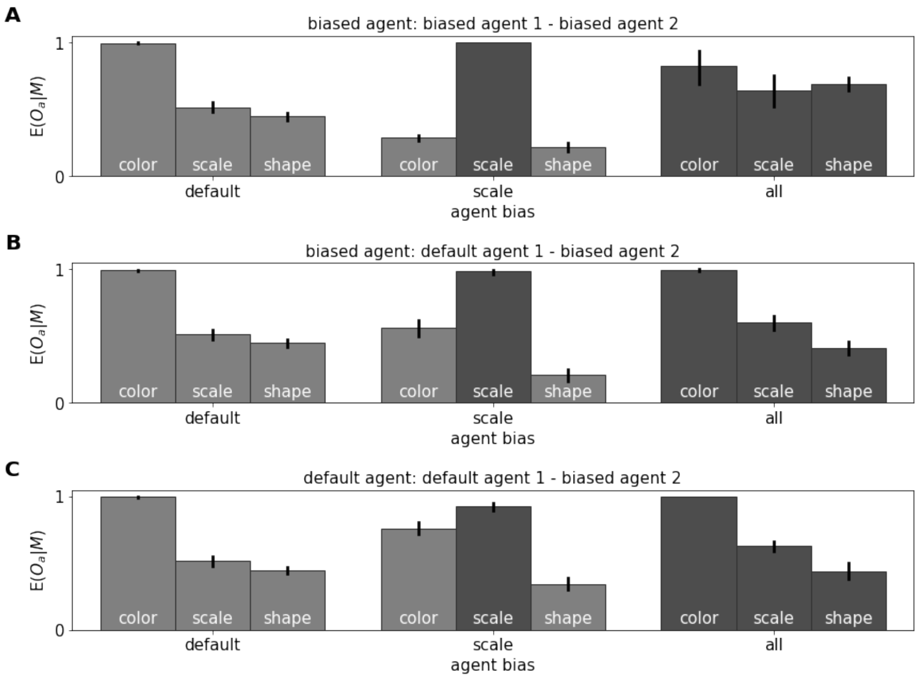

Fig 1. Effectiveness per attribute for different pairings of flexible-role agents. Pairings are (A) two agents with the same perceptual bias and (B)+(C) one biased agent and one DEFAULT agent. (B) shows the effectiveness scores for the biased agent and (C) the effectiveness scores for the DEFAULT agent in that mixed combination. The x-axis indicates the bias (of the biased agent or both agents). The bars are labeled with the attribute a used for calculating $E(O_a \mid M)$, with attributes enforced via label smoothing in dark gray. We report means and bootstrapped 95% CIs of ten runs each.

(ii) In the language learning scenario, the flexible-role agent corresponds to the receiver in a fixed-role simulation. Hence, we will only consider the language emergence scenario. The agents achieve average rewards between 0.955 and 0.967 on the test set. The attribute-wise RSA scores are shown in Fig 2, which corresponds to Fig 7 of the fixed-role agent simulations in the main manuscript. In analogy to Fig 8 in the main manuscript, we calculate the difference in general RSA scores before and after training. The RSA score of the DEFAULT agent improves from 0.439 before training to 0.536 (DEFAULT partner), 0.569 (SCALE partner), and 0.572 (ALL partner).

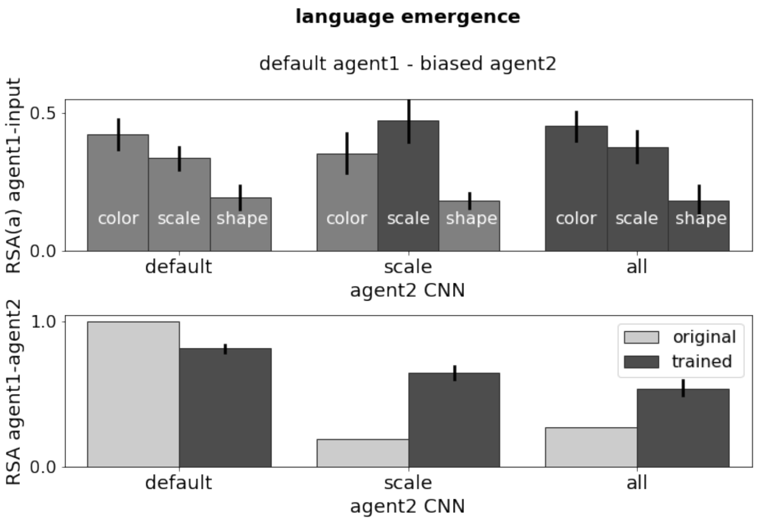

Fig 2. Influence of linguistic biases on perception. Shown are the effects of language emergence on a DEFAULT agent, when paired with agents of different visual bias conditions. The visual bias of the communication partner is shown on the x-axis. The top row shows the RSA scores between the DEFAULT agent's visual representations and each object attribute—indicated by the bar label—after training. The bottom row shows the RSA scores between the visual representations of the DEFAULT agent and those of its communication partner before (light gray) and after (dark gray) training. Reported are means and bootstrapped 95% CIs of ten runs each.

(iii) Fig 3 shows the test rewards for different combinations of flexible-role agents, which corresponds to Fig 9.A of the fixed-role agent simulations in the main manuscript. The rewards achieved by the flexible-role agents are slightly lower than those of their fixed-role counterparts. The game is already symmetric, so no additional calculations are necessary to perform a stable state analysis. Only the ALL bias is evolutionary stable, and this stability is significant as determined by pairwise comparisons of the CIs in the third matrix column.

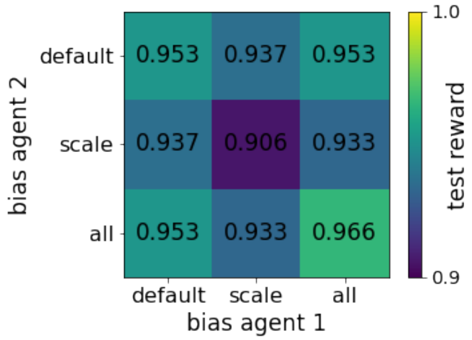

Fig 3. Mean reward on the test set for different combinations of two flexible-role agents. We ran 10 simulations for each combination.

In sum, across analyses, the findings from simulations with fixed-role agents generalize to simulations

with flexible-role agents.
