# Supplementary material for: Mutual influence between language and perception in multi-agent communication games
Source: PLoS Comput Biol. 2022 Oct 31;18(10):e1010658. doi: 10.1371/journal.pcbi.1010658 (PMC9648844; doi:10.1371/journal.pcbi.1010658)
Supplement: Attachment — Submitted filename: response_letter.pdf [file pcbi.1010658.s013.pdf]

Dear editors,

thank you very much for finding excellent reviewers for our manuscript. We would like to thank you and all reviewers for your extremely valuable feedback.

We have made extensive changes to the manuscript, including a thorough revision of the text, new simulations, and additional figures, to address all suggestions and concerns.

In particular, we have added substantial material to the discussion to address the concerns of Reviewer 1 that our simulations are too simple to make any claims about the interaction between language and perception in cognition. In short, we agree that our approach has limitations and address these limitations in the discussion. We also highlight that our setup is simple but therefore highly controlled and that it constitutes a significant step forward from earlier simulations.

The point about the evolutionary time scales, which is brought up by Reviewer 1 and Reviewer 3, has sparked fruitful internal debate. We have decided on a more general but at the same time also more cautious interpretation of our results. We have rewritten the introduction and the results to make clear that we focus on stable state analysis because it is compatible with many different interpretations of how stability might be achieved, including agent-internal and cross-generational optimization processes other than biological evolution.

To address the major points by Reviewer 3 we have added a wide range of simulations. A part of these simulations extends our setup to flexible-role agents and more-than-2 agent scenarios. Another part of these simulations addresses the role of the classification loss, especially when studying the effects of language on perception. Among others, we provide the results without classification. Importantly, the main findings could be confirmed in all these controls.

Finally, we have added references to additional literature provided by Reviewer 2 and Reviewer 3 in the introduction and the discussion.

We hope you agree that we have addressed the reviewers’ comments adequately and that you will consider the revised manuscript for publication in PLOS Computational Biology.

Kind regards,
Xenia Ohmer, Michael Marino, Michael Franke, Peter König

****

Reviewer #1

This paper addresses an interesting issue: the potential interactions between visual perception and language. And uses computational simulations in which agents are possess deep convolution neural networks, with the aim of building a model with theoretical implications for the potential mutual interdependence of language and perception. The simulations are well done, and the paper generally clearly written. My concern, though, is that the simulations and their results are not really sufficient to significantly address these broader issues in cognitive science at which they are aimed.

> Thank you very much for your feedback! As we understand it, the first part of your feedback concerns the simplicity of our setup (in different dimensions), and the second part of your feedback concerns the possibility that there is an evolutionary influence of language on perception. We will reply to these two points separately below.

As far as I can tell, the set of stimuli, and categories, is extremely simple (stimuli appear to vary on a small number of dimensions); and the computational problem of categorising these stimuli is close to trivial (i.e., the full power of deep learning is not required). For many purposes, such simplification absolutely fine; but when part of the objective is to understand the potential implications of communication on perception, I suspect it is not.

One reason is as follows: given the incredibly difficult challenge of processing natural images, the visual system is under enormous pressure to organise itself in a way that captures the complex hierarchical structure of those images. And then adding the additional selectional pressure to communicate specific messages could, potentially, pale into insignificance.

This problem is amplified by the fact that the visual brain is strongly constrained by the requirement to control the motor system, and to engage in complex interactions with the world (to know what is a potential predator, or what is edible), irrespective of communication. Again, the impact of the need to communicate may be dwarfed by the intense and continual pressure for the perceptual system to create representations interface naturally with action.

So the theoretical question of interest, when considering the human case in which language may provide additional constraints, has to be considered in this light. Could it be that the pressure of needing to communicate can be powerful enough to partially reshape the vision-internal constraints (and overcome action-relevant constraints)?

Demonstrating that there can, in principle, be an interaction between communication and categorisation just seems to low a bar - I think most computational modelers would assume that this is bound be possible in principle. The real question whether it happens in practice, given the actual informational and communicative constraints imposed by real vision and real language. Using an

approach similar to that outlined in the paper, it would surely be possible to explore this kind of question with deep neural networks, given that they are able to do reasonably good categorisation of real visual images, as well as to generate reasonably good natural language outputs. I think this would be much more interesting, and potentially a good contribution to the journal. Of course, drawing conclusions about the human case would still face the challenge that any such model may rather underestimate the degree to which the visual system requires help us navigate the everyday world, as well as communicate with each other (indeed, for a nonhuman animal, this is pretty much all the visual system has to do). But this would be the kind of issue that could be dealt with in discussion, perhaps.

The paragraphs above criticize that our setup is too simple in terms of images, categories, and more generally constraints on the visual system. We understand this argument and we think your criticism is—at least to some degree—justified. We still think that our work makes a valuable contribution. But thanks to your feedback (and the feedback from Reviewer 2) we are more nuanced in our claims, discuss our results more critically and point out directions for future work.

Our stimuli and neural network architecture may be simple but they constitute a significant increase in complexity compared to the stimuli and architectures used in earlier simulations studying the effects of category/language learning on perception (e.g., Cangelosi & Harnad, 2002; Lupyan, 2008; Westermann & Mareschal, 2014). There is a tradeoff between control/interpretability and complexity, and our implementation leans towards the former. Using objects that are composed of a fixed set of attributes and attribute values has several advantages. We can introduce selective visual biases via relational label smoothing, and we can quantify and compare visual and linguistic biases with respect to these attributes.

While our CNNs do not cover the complexity of biological vision, they rely on the same computational principles as larger CNNs that are currently state-of-the-art models of vision (e.g., Lindsay, 2021; Storrs, Kietzmann, Walther, Mehrer, Kriegeskorte, 2021), namely convolutional layers, hierarchical feed-forward processing, and training on a classification task. Although it remains to be shown, we believe that, qualitatively, the effects of language on the visual representations will persist in larger models.

Our proposal to use current deep learning architectures to study interactions between vision and language is novel. Our model is only a first implementation of this idea. As you and Reviewer 2 rightly criticize there are many functions of vision, and these functions imply different requirements and constraints on the visual system. We think that our work is an important first step. Future work can build on our simulations using state-of-the-art models trained on diverse natural images and categories.

Note, however, that even most state-of-the-art deep learning models in computational neuroscience capture only a few of the functional constraints on the visual system (let alone biological constraints, which is a separate discussion as you point out). For example, most models rely on classification, although classification is only one function of the visual system.

There are many ideas on how the correspondence between artificial and biological neural networks can be further improved by changing architectures, learning algorithms, input statistics, or training objectives (for overviews see Kietzmann, McClure, Kriegeskorte, 2019; Richards, Lilicrap, Beaudoin, Bengio, Bogacz, Christensen, et al., 2019). Another promising direction lies in deep reinforcement learning agents whose neural networks can incorporate constraints through "embodiment" and action (e.g., Clay, König, Kühnberger, Pipa, 2021). As of now, these agents are typically trained in highly specialized environments, though. We hope that our framework draws attention to interaction effects between language and perception (or other areas of cognition) in these research fields.

Finally, in direct response to the critique that "the impact of the need to communicate may be dwarfed by the intense and continual pressure for the perceptual system to create representations interface naturally with action" we would like to point out that there is not necessarily a strong contrast in kind between a pressure on the visual system from "acting in the world", on the one hand, and "communication", on the other. The kind of communicative scenario we consider here (a signaling game, as also used in theoretical biology) is essentially one in which a concrete action (the receiver's choice) performed in the real world yields a concrete payoff for either one or both agents, sender and receiver. However, we concede that it is plausible that pressure from single-agent actions and decision-making may outweigh pressure from these communication-based (inter)actions.

We made several changes to incorporate some of the discussion above in our manuscript:

- Based on the feedback from Reviewer 2, we specified that we are not studying the effect of language in general, but the effects of referential labels with denotations as sets of objects. Reference is arguably a core function of language and developed before more complex functions. You can find this specification in Paragraph 4 of the introduction.
- We added a subsection "limitations" to the discussion. In the first paragraph, we discuss the simplicity of our stimuli and categories. We explain that this simple setup provides us with experimental control in terms of inducing and quantifying visual and linguistic biases. In the second paragraph, we discuss the limited function of visual representations in our simulations (classification and communication). We conclude that our results likely overestimate the effects of language on vision.
- We added one paragraph at the beginning of the outlook to discuss possibilities for improving the correspondence between artificial and biological neural networks. We

conclude that these recent developments could be exploited to study interactions between language and vision under additional and hence more realistic constraints.

References

- Cangelosi A., Harnad S. (2000) The adaptive advantage of symbolic theft over sensorimotor toil: Grounding language in perceptual categories. Evolution of Communication, 4, 117-142.
- Clay, V., König, P., Kühnberger, K. U., Pipa, G. (2021) Learning sparse and meaningful representations through embodiment. Neural Networks, 134, 23–41
- Kietzmann, T. C., McClure, P., Kriegeskorte, N. (2019). Deep neural networks in computational neuroscience. Available from: https://oxfordre.com/neuroscience/view/10.1093/acrefore/9780190264086.001.0001/acrefore-9780190264086-e-46
- Lindsay, G. W. (2021). Convolutional neural networks as a model of the visual system: Past, present, and future. Journal of Cognitive Neuroscience, 33(10), 2017-2031.
- Lupyan, G. (2012). What do words do? Towards a theory of language-augmented thought. In B. H. Ross (Ed.), The Psychology of Learning and Motivation (Vol. 57, pp. 255–297). Academic Press.
- Richards, B. A., Lilicrap, T. P., Beaudoin, P., Bengio, Y., Bogacz, R., Christensen, A., et al. (2019). A deep learning framework for neuroscience. Nature Neuroscience, 22(11), 1761-1770
- Storrs, K. R., Kietzmann, T. C., Walther, A., and Mehrer, J., and Kriegeskorte, N. (2021). Diverse deep neural networks all predict human inferior temporal cortex well, after training and fitting. Journal of Cognitive Neuroscience, 33(10), 2044-2064.
- Westermann, G. & Mareschal, D. (2014). From perceptual to language-mediated categorization. Philosophical transactions of the Royal Society of London B, 369(2012039)

A final more specific theoretical point is that I think the third theoretical goal of the paper, concerning the possible coevolution of human vision and language needs very careful thought. The visual system is very old; and language is both very new by comparison, but also extremely rapidly changing, and, for that matter, the categories used across languages are themselves very variable (just think of the extraordinary variability of colour categories, for example).

So any story in which there is substantial coevolution of the visual system to linguistic categories is going to be very hard to get off the ground--- it would have to assume that there are certain categories that are extremely stable across languages and across time, and old enough to have a substantial influence on genes underlying the neural organization of vision. And this certainly will not

be true of many of the categories that we use in everyday life, so many of which refer to an artificial world of objects and institutions that didn't even exist until the last few centuries (think of chairs, pens, pizzas, offices, etc).

This would seem to imply that language is unlikely to play a major role in shaping vision through biological evolution. It is much more credible that the need to learn the categories of a specific language might, within the lifetime of the individual, influence the structure of the visual system at least to some extent. After all, this is clearly true of learning to read--- the specific neural machinery for recognising letters is biologically "wired in" but obviously not through processes of biological evolution (as literacy is so recent; and the 'machinery' is specific to particular scripts/languages).

> **Addressed**. Thank you for voicing this concern. Your arguments have motivated us to reconsider the interpretation of our evolutionary analysis. Specifically, what we show is that there might be pressure for the specialization of the perceptual system to the linguistic environment. We do not claim that this is exactly what happens in the biological example, and we use more precise phrasing in the manuscript to delineate the sense in which the evolutionary analysis should or should not be interpreted as pertaining to biological evolution.
>
> For the reasons you mention, it has been doubted that language could have influenced the brain, and therefore also visual perception, through a process of biological evolution (Christiansen & Chater, 2008). However, as the peer commentary on the cited article shows, the matter is highly debated. A major problem lies in the fact that details about the evolution of the visual system as well as the evolution of language are unknown. The (macaque) monkey visual system is often and successfully taken as a model system for the human visual system. The mainstream view is that the two visual systems share many characteristics but are not identical (Orban, van Essen, Vanduffel, 2004; Rapan et al., 2021). Further, as research is guided by our knowledge of the monkey visual system and largely different methods are used in experimental investigations of the two species (invasive recordings vs. fMRI), a precise estimate of how similar or different the two systems are is difficult. Furthermore, the origin of language is an unresolved question, and even its discussion has been banned on one occasion (Hauser et al., 2014). To make matters more complex, individual development is related to ontogenetic development (Hill et al., 2010). Overall, it is difficult to estimate the relative change of perception during the evolutionary emergence of language. Therefore, we take a conservative approach and refrain from specific claims on whether language development was or was not accompanied by substantial evolutionary changes in the brain.
>
> These aspects are shortly discussed in the revised manuscript under Discussion - Evolutionary analysis.
>
> However, the evolutionary stable state (ESS) analysis can be interpreted more generally. The revised version of the paper includes a completely rewritten section on the evolutionary

analysis (see Introduction (iii)), stressing that the choice of an analysis in terms of evolutionary stability is precisely to *avoid* commitment to a specific process of optimization of evolution. ESSs are the (locally) asymptotically stable rest points of the replicator dynamic (Taylor & Jonker 1978, Hofbauer & Sigmund 1998). The replicator dynamic, in turn, is a rather encompassing high-level formalization of a wide variety of agent-internal optimization processes, be they cross-generational (cultural evolution, (asexual) reproduction (Sandholm 2010)), or within-generational (simple forms of reinforcement learning (Börgers & Sarin 1997), imitation-based dynamics (Sandholm 2010, Franke & Correia 2016)). Hence, the evolutionary analysis substantiates the claim that an agent's perceptual system might be optimized over time to the affordances of their environment also by the kind of linguistic interactions we look at, without specifying the nature and time scales of this optimization.

References

- Christiansen, M. H., & Chater, N. (2008). Language as shaped by the brain. Behavioral and brain sciences, 31(5), 489-509
- Börgers, T., Sarin, R. (1997). Learning through reinforcement and replicator dynamics. Journal of Economic Theory, 77(1), 1-14
- Franke, M., Correira, J. P. (2018). Vagueness and imprecise imitation in signaling games. The British Journal for the Philosophy of Science, 69(4), 1037-1067
- Hauser, M. D., Charles, Y., Berwick, R. C., Tattersall, I., Ryan, M. J., Watumull, J., Chomsky, N., Lewontin, R. C. (2014). The mystery of language evolution. Frontiers in Psychology, 5(401), 1-12
- Hill, J., Inder, T., Neil, J., Dierker, D., Harwell, J., van Essen, D. (2010). Similar patterns of cortical expansion during human development and evolution. Proceedings of the National Academy of Sciences (PNAS), 107(29), 13135-13140
- Hofbauer, J., Sigmund, K. (1998). Evolutionary games and population dynamics. Cambridge, MA: Cambridge University Press
- Orban, G. A., van Essen, D., Vanduffel, W. (2004). Comparative mapping of higher visual areas in monkeys and humans. Trends in Cognitive Sciences, 8(7), 315-324
- Rapan, L., Niu, M., Zhao, L., Funck, T., Amunts, K., Zilles, K., Palomero-Gallagher, N. (2022). Receptor architecture of macaque and human early visual areas: not equal, but comparable. Brain Structure and Function, 227, 1247–1263
- Sandholm, W. H. (2010). Population games and evolutionary dynamics. Cambridge, MA: MIT Press
- Taylor, P. D., Jonker, L. B. (1978). Evolutionary stable strategies and game dynamics. Mathematical Bioscience, 40(1-2), 145-156

Overall, this is well conducted work, and a well written manuscript; but it’s overall theoretical contribution seems not to be sufficient for publication in PLOS Computational Biology.

****

Reviewer #2

I was very impressed by Ohmer et al's manuscript. It demonstrates excellent scholarship — bringing in much of the key literature. For example, I made a note on p. 4 indicating the high relevance of Cangelosi et al's. now fairly old work on "sensorimotor toil" vs "symbolic theft" and was impressed to see it cited later on! I've done some conceptually similar modeling as part of a larger "language augmented cognition" project, e.g.,

Lupyan, G. (2012). What do words do? Towards a theory of language-augmented thought. In B. H. Ross (Ed.), The Psychology of Learning and Motivation (Vol. 57, pp. 255–297). Academic Press.
http://www.sciencedirect.com/science/article/pii/B9780123942937000078
(http://sapir.psych.wisc.edu/papers/lupyan_2012_languageAugmentedThought.pdf)

but the authors go way beyond the toy models presented in that work.

The manuscript is also very clear and thorough in its presentation of methods and results. This is an impressive project especially considering the student first authors. Below are some relatively minor comments.

Thank you very much for your positive evaluation and feedback! We will reply to the detailed points below.

1. The many functions of vision

"To study the influence of language on perception, we allow agents to adapt their visual representations (CNN weights) while playing the communication game." — this is a fine approach, but the authors should be more explicit that vision has many functions; yielding representations that we can (however approximately) talk about, is just one function. Hence, we are highly adept at distinguishing small differences in hue even though we cannot easily put them in words. The categorical effects on perception that we do see (especially in vision) are rather slight, e.g., categorical color perception goes away entirely in some tasks, eg.,

Witzel, C., & Gegenfurtner, K. R. (2015). Categorical facilitation with equally discriminable colors. Journal of Vision, 15(8), 22–22. https://doi.org/10.1167/15.8.22

and while language enhances categorical perception, e.g., as we describe in this paper:

Forder, L., & Lupyan, G. (2019). Hearing words changes color perception: Facilitation of color discrimination by verbal and visual cues. Journal of Experimental Psychology: General, 148(7), 1105–1123. https://doi.org/10.1037/xge0000560

in the paper above, you'll note that on the no-label trials people's discrimination is about equal within and between categories.

So, if an agent's only goal is to communicate about a discrete set of categories, you'd expect perception to just represent things categorically. But because categorization/naming/communication are just one goal of perception, perceptual systems continue to represent "category irrelevant" properties. Because in your model the perception is *for* communication, the effects of having to communicate about this or that dimension likely over-estimate the kinds of effects of language on perception one tends to observe.

> **Done**. This is a very important point. Indeed the functionality of the agents' visual systems is very narrow and stands in contrast to the plurality of our interactions with the world relying on visual perception. Your feedback is related to that of Reviewer 1, who points out that the visual system is subject to various constraints, for example, the requirement to control the motor system. By implementing only a subset of the functionality of the visual system, we do not account for all the pressures/constraints on visual representations.
>
> We addressed this point by adding two paragraphs to the discussion. The first paragraph lies right before the outlook section (second paragraph under "limitations") and emphasizes the reduced functionality of vision in our simulations. It also points out that this will likely lead to an overestimation of the influence of communication on visual representations in our model. The literature you provided was very helpful. We included it here, to make clear that the influence of language on perception can be transient and conditional, which is not captured by our model. Finally, we conclude that a more complex model might account for these nuances. This idea is fleshed out in the second paragraph, which follows right afterward (first paragraph under "outlook"). We review some recent developments in computational neuroscience to show that models going beyond feed-forward architectures and beyond classification could be used to account for additional constraints/pressures on the visual system.

2. Enhanced perception?

"Enhanced perception of relevant features is evolutionary stable. " (p. 23).

I would have liked to see some discussion of *what* the authors think is being enhanced. If the models here are taken as very roughly representing human visual systems, what about perception is being enhanced? And how "perceptual" is it? For example, "enhanced perception" can be achieved

purely through attentional modulation. Although attention largely works by altering perceptual representations through the processing hierarchy, there is a difference between “better” perception due to a high level attentional selection mechanism vs. a dynamic retuning of receptive fields of primary sensory neurons. If you think the influence of communication/language on perception is not attentional (the current models after all lack attentional mechanisms), are you thinking of a more permanent “warping” of low-level visual representations? Or are the differences more likely to be observed higher up in the processing hierarchy? If the latter, are these changes still “perceptual” (rather than more decisional)? (Many people care about these differences, e.g., as they relate to questions about cognitive penetrability of (especially low-level) perception).

Partially addressed. We understand that this is an important point. We did not discuss it because we think that our model does not really allow us to make any statements about which part of perception, or perceptually-informed decision making, is affected by language in human cognition.

Our model is relatively small, with only two convolutional and two-fully connected layers. In addition, it simulates a reduced function of vision in terms of classification (related to your point “the many functions of vision”). Without a significant increase in architectural and functional complexity, an analysis of the depth of the effects of communication on visual representations in our model does not warrant conclusions about mechanisms in the human visual system.

So far, our model shows that representations informed by visual perception can change through language use. However, we are aware of the task-sensitivity of the influence of language on perception (see, e.g., your reference to Witzel & Gegenfurtnerl, 2015, above) and additional nuances, for example, lateralization effects (Regier & Kay, 2009). We would therefore like to avoid claims about whether the language-induced representational changes in our model are relatively permanent, or about the time scales necessary to learn, overwrite, or unlearn them.

In sum, future work is needed to address this point. Using a more complex model of vision could help to make quantitative claims about representational change, including statements about penetration depth. More complex architectures could further be equipped with attention mechanisms (which we do not exclude out of principle). Our model captures offline effects of language on perception that arise through learning. By adding an attention mechanism, the model could be extended to online effects, too.

In the revised manuscript We discuss this in the second paragraph under Discussion-Limitations.

References

- Lupyan, G., Abdel Rahman, R., Boroditsky, L., Clark, A. (2020). Effects of Language on Visual Perception. Trends in Cognitive Sciences, 24(11), 930-944
- Regier, T., Kay, P. (2009). Language, thought, and color: Whorf was half right. Trends in Cognitive Sciences, 13(10), 439-446

3. Some additional relevant literature

> Thank you very much for pointing out these papers to us. We read them with great interest! Given the relevance of these papers, we included references in the manuscript (see details below).

If you are not familiar with it, you may be interested in the following papers:

Ozgen, E., & Davies, I. R. L. (2002). Acquisition of categorical color perception: A perceptual learning approach to the linguistic relativity hypothesis. *Journal of Experimental Psychology-General*, *131*(4), 477–493.

> We rewrote the two paragraphs discussing our findings on the effects of language on perception (lines 567-592 in the original submission) to include information from this paper as well as information from the paper on the conceptual grouping effect below. We felt that it made sense to restructure the former two paragraphs into three new paragraphs which include the additional references without disturbing the reading flow. The first paragraph describes our findings and references the two papers as related empirical work. The second paragraph provides information about the neural network simulations by Harnad, Hanson, and Lubin, as well as Cangelosi and Harnad. The last paragraph adds information on the representational alignment between communication partners.

You cite some work by Luc Steels, but this key paper is an omission:

Steels, L., & Belpaeme, T. (2005). Coordinating Perceptually Grounded Categories Through Language: A Case Study for Colour. Behavioral and Brain Sciences, 28(04), 469–489. https://doi.org/10.1017/S0140525X05000087

> **Done**. Our submission did cite this paper. You can find the reference in the first paragraph of the Related work section. If you have another place in mind, please let us know.

Finally, you may be interested in this recent paper where we investigated the effects of different types of labels on DNN visual representations and fit to human behavior:

Ahn, S., Zelinsky, G. J., & Lupyan, G. (2021). Use of superordinate labels yields more robust and human-like visual representations in convolutional neural networks. Journal of Vision, 21(13), 13. https://doi.org/10.1167/jov.21.13.13

> This is very interesting work, and there are methodological similarities between training on labels that provide taxonomic structure and relational label smoothing. E.g., by spreading some of the target weight onto other red objects, information about a superordinate category "red" is provided to the network. Similarly, when training on ImageNet, for each dog, some target weight could be spread to all other classes of dogs, all classes of animals, etc. We think the paper fits nicely into our new first paragraph of the outlook section, which addresses ideas on how to bridge the gap between biological and artificial neural networks for vision. We included it in our list of examples and used the opportunity to draw a connection to relational label smoothing.

With regard to this point, "supervised learning of symbolic object descriptions warped these similarity spaces even further, leading to increasingly categorical perception", you may be interested in this empirical paper:

Lupyan, G. (2008). The conceptual grouping effect: Categories matter (and named categories matter more). *Cognition*, *108*(2), 566–577.

> See our comment under the suggested paper by Özgen et al. We rewrote the two paragraphs discussing our findings on the effects of language on perception (lines 567-592 in the original submission) to include information from both your paper and the paper by Özgen et al.

Other comments:

a. "Our simulations mirror several empirically observed phenomena: labels group together visually similar objects, " — it may be worth clarifying what kinds of labels you are thinking of. This is true for labels like "circle" and "bowl" and "striped". It's not so true for arguably the bulk of language. For example, none of the words I am using in this sentence really have anything to do with perceptual properties. And much of the language we use — especially among adults — has this property. We talk about social relationships, plans, desires, things removed in time — the categories denoted by these words can't really be described in terms of perceptual features (at least not in any straightforward way).

> **Done**. Thanks for pointing this out. Now, we make clear that we talk about *referential labels* with denotations as sets of real-world objects.

As the Author summary should not be technical, we picked up your examples and changed the sentence there to: “[...] labels for concrete objects and properties (e.g., ``striped'', ``bowl'') group together visually similar objects [...]”.

Later in Paragraph 4 of the Introduction, we go into more detail:
“[...] By choosing this kind of game, we study the emergence and effects of *referential labels*, with denotations as sets of real-world objects. Reference is arguably a core function of language around which more complex functions are organized (Jackendoff, 1999). [...]”

-Gary Lupyan

****

Reviewer #3

The authors explore the influences of communicative interactions on perception and the influences of perceptual biases on the emergent communication protocols. The authors use communication game simulations with two agents. They find that agents' perception adapts to the communication-relevant attributes, that emergent communication is biased towards perceptually enhanced dimensions, and that possessing a communication-relevant perceptual bias presents an evolutionary advantage.

Ohmer and colleagues' approach is novel, and it introduces an exciting new direction of research on both 1) language evolution and 2) effects of language on perception. So far, the area of language influences on perception has focused on one-agent simulations and experiments. Simulations of language evolution, on the other hand, have mainly used the agents that have no access to perceptual properties of the objects, or the agents' whose perception is not adaptive. This study is methodologically rigorous and extremely well done. The authors' main conclusions are overall well substantiated by their experiments. The paper is well written. This paper presents a complete investigation of the target questions, so I only have suggestions regarding authors' presentation of the approach and interpretation of the results. I am happy to recommend this paper for publication in PLOS Computational Biology.

> Thank you very much for your positive evaluation and feedback! We will reply to the detailed points below.

MAJOR COMMENTS

1. Generalizability. The authors investigate two-way influences of perceptual biases and communication by using two-agent simulations whose communicative roles (speaker and listener) are fixed. This limitation in the approach does not undermine the originality and rigor of this work, but it may limit authors' conclusions: for example, it has been shown that many results from two-agent and fixed-role simulations are not guaranteed to generalize to "more than 2"-agent cases. I recommend the authors to reflect on the generalizability of their results to "more than two"- and "flexible role"-agents (each agent learns to both speak and listen) scenarios in the discussion section.

> **Done**. Thanks for pointing this out. We were very curious ourselves how the system would generalize to other setups. Apart from adding a paragraph in the discussion to address your concern, we provide additional simulations with flexible-role agents as well as 4 agents (two senders and two receivers). It turns out that these alternative setups yield the same findings as the original 2-agent fixed-role simulations. While, of course, many more setups are

conceivable (e.g. further increasing the number of agents), we conclude that our findings are not tied to a special case but seem to reflect more general patterns of language-perception interactions in multi-agent communication.

The new paragraph in the discussion is called “Flexible-role agents and populations”. It gives a high-level overview of the problem and the results of our additional simulations. Details about the methods and results of the additional simulations can be found in the supplementary material (S5 Appendix and S6 Appendix). In particular, we limit the additional simulations to the *default*, *scale*, and *all* conditions because they cover the main types of perceptual biases in the original simulations: no manipulation (*default*), bias for a single attribute (here *scale*), bias for all attributes (*all*). For both the flexible-role and the 4-agent simulations, we repeat the three analyses (i) influence of perception on language, (ii) influence of language on perception, and (iii) evolutionary analysis, and generate figures that correspond to the original figures in the Results section for comparison.

2. Classification confounds. On page 11 (lines 201-205), the authors describe adding classification loss to the agents’ training to avoid their perceptual systems from “deteriorating” when fully adapting to the communication task. I would appreciate it if the authors clarify why such “hyper-adaptation” presents a problem for their experiments, as their target question is perceptual adaptation in response to communicative pressures, at least in experiment 2. More importantly, I expect that adding the classification task does not only attenuate the effects of communication on perception (as the authors note), but it also creates perceptual effects on its own (e.g. potential bias for representing color rather than shape or scale). I recommend the authors to explicitly note this for all the effects that can be potentially attributable to the classification task in experiments 1 and 2 (I see that this confound has been explicitly addressed for experiment 3). For example, on page 20 (lines 322-323), the authors describe one (potential) effect of the classification subtask: “In the default condition (left) the messages are strongly grounded in object color, which can be attributed to the inherent color bias of the default CNN” (this alternative, classification task-based, interpretation might also require authors to correct the discussion in lines 557-560)

Done. We agree that the role of the classification task has not received enough attention in the manuscript. We made several textual changes to the manuscript and added additional simulations to address this point.

1) Classification during pre-training (Experiments 1, 2, and 3)

To begin with there are good reasons for using the classification objective during pre-training. Our agents have a vision module, which is supposed to simulate visual processing, and a language module, which is supposed to simulate linguistic processing. The function of the visual system is reduced to object recognition (classification). This reduction constitutes a

severe simplification (see feedback from Reviewer 1 and Reviewer 2) but is not uncommon. In fact, many DNN models of vision are trained on a classification objective, which—using more complex input images—seems to capture activations in the visual cortex relatively well (see e.g., Lindsay, 2021; Storrs, Kietzmann, Walther, Mehrer, Kriegeskorte, 2021). Note also, that simulating vision any other way, for example as an image reconstruction process, would also induce task-specific effects.

⟶ We pick up this discussion briefly in the new subsection "Limitations" in the discussion.

The question remains, whether classification during pre-training has any undesirable or confounding effects on the emerging language. Importantly, we quantify the visual biases and the resulting linguistic biases. Hence, the color bias in the *default* condition is not a confound. We use the default labels, we find a visual *color* bias, and we find that a linguistic color bias emerges as a result. Our simulations demonstrate systematically that differences in representational similarities (regardless of how they come about) lead to differences in communication.

Still, in our simulations, the categories of the classification task and the reference game are aligned. A control simulation is helpful to understand what happens if that is not the case. We use the control simulation from Experiment 3, combining different relevance conditions (color irrelevant, scale irrelevant, shape irrelevant) with different bias conditions (color-scale, color-shape, scale-shape), and quantify the linguistic bias (effectiveness scores) for each combination. The results show again, that perceptual biases are reflected in linguistic biases. In addition, they show that irrelevant attributes are not communicated regardless of the agents' bias.

⟶ We provide the linguistic analysis of these mixed-bias simulations in the Supporting information (S5 Fig).

2) Classification during language emergence (Experiment 2)

There are also good reasons for keeping the classification objective in the simulations studying the effects of language on perception. Once it is decided that vision is simulated as (and reduced to) object recognition, it is strange to remove this classification task in the language emergence simulation. When learning to communicate, the visual system still fulfills all its functions. We keep the classification loss, to simulate that visual representations have other purposes than informing communication. As discussed by Reviewer 1 and Reviewer 2, it would actually be desirable to increase the number of functions that the visual system fulfills rather than decrease them.

The agents in our simulations tend to find suboptimal solutions, i.e. they group together multiple objects based on a single attribute. This behavior simplifies communication but comes

at the cost of a decrease in reward, and has been observed elsewhere (e.g., Chaabouni, Kharitonov, Bouchacourt, Dupoux, & Baroni, 2021), as we explain in the discussion. In the case of these "suboptimal" solutions, the classification loss guarantees that the visual representations still contain information that is sufficient to classify each object. Our results are rather interesting in showing that the agents' linguistic bias is reflected in their visual representations *even though* the visual system does more than generate representations for communication.

⟶ We try to convey this argument briefly by changing the "General setup" paragraph in "Materials and methods" to: "[...] When learning to communicate, visual representations may adapt but they are still constrained by the functions of the visual system. In our case, this function is limited to object recognition (classification). To ensure that the agents' perceptual ability does not deteriorate to processing only aspects relevant to the communication game, training on the classification task used for pretraining continues. [...]"

Still, it would be useful to have the results without the additional classification loss for comparison. We ran these simulations for the *default*, *scale*, and *all conditions* (see above). The main findings can be confirmed also without classification: If the agents learn a language from a biased agent (e.g. scale) or they develop a language together with a biased agent, the linguistic bias translates to a visual bias, in that the RSA score for the corresponding attribute increases. However, the classification loss has a moderating effect on the RSA scores as it constrains the visual representations to capture differences between the values of attributes that do not conform to the linguistic bias. Thus, it has the predicted effect of keeping the visual representations from deteriorating to capture only aspects that are relevant to the communication game. As the agents discriminate between fewer objects in communication than in classification (communication but not classification is suboptimal), visual representations of the three attributes (general RSA scores) do not improve without the classification loss.

(By the way, it turns out that the *color* bias is inherent to the architecture/ input representation and does not come from the classification task. In the language emergence simulations without classification loss, the color bias even *increases* because it is no longer constrained by the classification task.)

⟶ We include an overview of these results in the Section "Influence of language on perception"- paragraph "The role of classification". Detailed descriptions of methodology and results can be found in the supplementary material (S3 Appendix).

References

- Chaabouni, R., Kharitonov, E., Bouchacourt, D., Dupoux, E., Baroni, M. (2020). Communicating artificial neural networks develop efficient color-naming systems. Proceedings of the National Academy of Sciences (PNAS), 118(12), e2016569118
- Lindsay, G. W. (2021). Convolutional neural networks as a model of the visual system: Past, present, and future. Journal of Cognitive Neuroscience, 33(10), 2017-2031.
- Storrs, K. R., Kietzmann, T. C., Walther, A., and Mehrer, J., and Kriegeskorte, N. (2021). Diverse deep neural networks all predict human inferior temporal cortex well, after training and fitting. Journal of Cognitive Neuroscience, 33(10), 2044-2064.

MINOR COMMENTS

When introducing or discussing the effects of perceptual biases on communication, I recommend the authors to refer to Morten Christiansen's work to better frame their contribution within the current hypotheses on the drivers of language evolution:

Christiansen, M. H., & Chater, N. (2008). Language as shaped by the brain. Behavioral and brain sciences, 31(5), 489-509.

> **Done**. This is indeed a useful resource. Among others, it helped our internal discussion about the evolutionary analysis, which was triggered by Reviewer 1. We have included a reference in the subsection Discussion-Evolutionary analysis.

Lines 22-24, page 3: "The formation of linguistic expressions is strongly influenced by perception, not only for concrete concepts like colors [17] but also abstract ones [18]". This sentence is confusing. Do the authors mean perception for concrete "attributes" (=color)? How does "perception" of abstract "concepts" look like? I recommend rewriting or clarifying this sentence.

> **Done**. We rephrased the sentence to "Expressions for concrete concepts like colors depend on perception [17]. But also abstract concepts can be understood and represented via metaphoric mappings to concrete concepts grounded in sensorimotor experience, for example in reasoning about time as a moving object ('The time will come when …', 'Time flies …') [18]."

Line 76, page 5: authors use the term "natural selection": I am not sure if it is appropriate in this context. Authors' evolutionary simulations indeed reveal the "selection" pressures, but such pressures can be realized in either "natural" or "cultural" evolution.

> **Done**. Thank you very much for this pointer. You are right, we do not intend to commit ourselves to biological evolution. (For details you can check out our response to the feedback from Reviewer 1.) We changed the document throughout, including at the position you point out here, to remain agnostic about the type of evolutionary process we investigate. In particular, we entirely rewrote the section about the evolutionary analysis in the Introduction.

Caption to Fig.1, page 8: “Simultaneously, the receiver’s vision module processes the images of the target and the distractor images.” The use of “simultaneously” is confusing here: do the authors mean that all the images were processed at the same time? I assume this is not the intended interpretation as the receiver’s vision module seems to only have one processing stream.

> **Done.** You are right. The sentence is misleading as the receiver’s vision module has only one processing stream. We changed the sentence to: “In addition, the target and the distractor images are processed by the receiver's vision module.”

Fig. 2, page 10: This figure might be very helpful for understanding relational smoothing, but it was difficult for me to understand it. I recommend the authors to revise it or its caption to make it more accessible.

> **Done**. We made several changes to present the information more clearly. In the old version of Fig.2 parts A and B illustrate how perceptual bias can be *generated* with relational label smoothing, while parts C and D illustrate how perceptual bias can be *quantified* with RSA. To present the information more clearly we used this natural division and split the figure into two figures: Parts A and B are shown in Figure 2, and parts C and D have been moved to a new Figure, which is now Figure 4. In addition, we changed the caption of Figure 2, such that it contains much more detailed explanations. We adapted the text slightly to account for these changes.

Fig. 6, page 21: The bars and labels on this figure are confusing. Do the x-axes represent sender/receiver CNN biases’ conditions?

> **Done**. We changed the x label from “sender CNN”/ “receiver CNN” to “sender CNN bias”/ “receiver CNN bias”. In addition, we improved the figure caption. We now state explicitly that the x-axis shows the CNN bias of the communication partner, and that the bar labels (in the top row) indicate the attribute for which the RSA score is calculated. More generally, we tried to explain more clearly what is shown in each row and column.

Lines 642-643, page 30: this sentence is not clear. What kind of “interaction” do the authors have in mind?

> **Done**. Thank you, we added an explanatory sentence: “In fact, it seems likely that all interfaces between cognition and language are mutually adapted towards optimal interaction in the environments we face [84], such that language can guide the acquisition of cognitive representations from experience, and in turn, can be used to structure and exchange these experiences [85].”

Marina Dubova
