# Supplementary material for: Mutual influence between language and perception in multi-agent communication games
Source: PLoS Comput Biol. 2022 Oct 31;18(10):e1010658. doi: 10.1371/journal.pcbi.1010658 (PMC9648844; doi:10.1371/journal.pcbi.1010658)
Supplement: Attachment — Submitted filename: response_letter.pdf [file pcbi.1010658.s014.pdf]

Dear editors,

We would like to thank you for reviewing our paper again and for forwarding our work to the reviewers. We improved the manuscript based on the comments from Reviewer 2. In particular, we changed a sentence in the introduction as suggested, and we added a few sentences to the "General setup" (p.10) to clarify the role of the classification loss. Reviewer 1 and Reviewer 3 did not suggest any additional changes.

Below you can find a more detailed point-to-point response.

Kind regards,

Xenia Ohmer, Michael Marino, Michael Franke, and Peter König

Reviewer #1: This is high quality work. My main concern on the first round was that the contribution is not quite substantial enough to be useful - because the basic pattern of results feels more or less inevitable (if you impose a communicative task, a 'visual system' will be shaped by it); the real question, to me, is the relative informational demands of vision-for-action and vision-for-communication in natural language.

My concern remains - but in the light of the feeling from the other reviewers that this is a sufficient contribution, and perhaps a stimulus to further work, I am happy to recommend acceptance. I do hope the authors (or another team) pick up this line of work using more realistic images, to address the informational question more thoroughly in the future.

I appreciate the adjustment to the paper regarding the evolutionary picture.

> Thank you very much for recommending our paper! We, too, hope that there will be follow up work with more realistic images.

Reviewer #2: I commend the authors for such a thorough revision. I only have a few remaining comments:

> Thank you very much for your additional comments! We have followed all your suggestions.

1. “ language manipulates judgments of perceptual similarity by imposing categorical structure [20]” — the better word choice here would be “affects” in place of “manipulates”. More importantly though, the cited work is quite old (and flawed) and has been superseded by better controlled studies that get at effects of language on visual processing rather than higher-level judgments e.g.:

Winawer, J., Witthoft, N., Frank, M. C., Wu, L., Wade, A. R., & Boroditsky, L. (2007). Russian blues reveal effects of language on color discrimination. *Proceedings of the National Academy of Sciences of the United States of America*, *104*(19), 7780–7785.

Winawer, J., Witthoft, N., Frank, M. C., Wu, L., Wade, A. R., & Boroditsky, L. (2007). Russian blues reveal effects of language on color discrimination. *Proceedings of the National Academy of Sciences of the United States of America*, *104*(19), 7780–7785.

Forder, L., & Lupyan, G. (2019). Hearing words changes color perception: Facilitation of color discrimination by verbal and visual cues. Journal of Experimental Psychology: General, 148(7), 1105–1123. https://doi.org/10.1037/xge0000560

We changed the sentence from “language manipulates judgments of perceptual similarity” to “language affects perceptual processing”. In addition, we used the two references suggested by you instead of the reference to Kay (1984).

2. I would like the authors to further clarify how they think of classification loss. Here is where I am coming from. In the case of naming an object, the category comes from language. Where are the categories that are being used in the non-communicative classification task coming from (conceptually)? I.e., when you write “ In the case of these “suboptimal” solutions, the classification loss guarantees that the visual representations still contain information that is sufficient to classify each object” — classify as what, exactly?

We added the following explanation to the “General setup” on p. 10.

“Categories do not have to originate from language. Categories can also be formed through interactions with the world, and nonhuman animals as well as preverbal human infants can learn categories (Sloutsky, 2017). Of course, these categories can still be lexicalized later on. The classification task is motivated by this ability to form categories through interactions with the world. While we do not explicitly model such interactions we assume they take place nonetheless.”

References:

Sloutsky, V. M. & Deng, W. (2019). Categories, concepts, and conceptual development. *Language, Cognition and Neuroscience*, *34*(10), p. 1284-1297

-Gary Lupyan

Reviewer #3: The revised version of the manuscript has further improved an already excellent work. All the reviewers' comments seem to be addressed, either completely or in essence. My own concerns are fully resolved -- I especially appreciate authors' efforts to further explore the generalizability of their results with additional simulations. I am looking forward to recommending this work to my colleagues as a reference, once it is published.

Thanks again for your feedback and for recommending our paper for acceptance!
